# MyD88 restricts dysbiosis-mediated inflammation in filaggrin deficient skin

**DOI:** 10.1371/journal.pone.0353968

**Published:** 2026-08-14

**Authors:** Meng-Jen Wu, Advaitaa Ravipati, Yu Wang, Laine E. Feller, Alyssa Chu, Rishi Damarla, Zhiyang Li, Lam C. Tsoi, Ryleigh Griffin, Peng Hou, Hai Liang, Raif Geha, Julia A. Segre, Heidi H. Kong, Johann E. Gudjonsson, Nathan K. Archer

**Affiliations:** 1 Department of Dermatology, Johns Hopkins University School of Medicine, Baltimore, Maryland, United States of America; 2 Department of Dermatology, University of Michigan, Ann Arbor, Michigan, United States of America; 3 Dermatology Branch, NIAMS, NIH, Bethesda, Maryland, United States of America; 4 Division of Immunology, Children’s Hospital and Department of Pediatrics, Harvard Medical School, Boston, Massachusetts, United States of America; 5 Translational and Functional Genomics Branch, NHGRI, NIH, Bethesda, Maryland, United States of America; INSERM, FRANCE

## Abstract

Atopic dermatitis (AD) is a common inflammatory skin disease associated with epidermal barrier dysfunction, immune dysregulation, and microbial dysbiosis. Loss-of-function mutations in filaggrin, a critical epidermal protein, represent the strongest genetic risk factor for AD and result in compromised skin barrier integrity and altered immune responses. MyD88 is an adaptor protein essential for TLR and IL-1 receptor signaling, with dual roles in promoting inflammation and regulating immune tolerance. However, the function of MyD88 in maintaining skin homeostasis in the context of filaggrin deficiency remains unclear. Here, we used filaggrin-deficient (ft/ft) mice crossed with MyD88 knockout mice (ft/ft^MyD88-/-^) to investigate the immunological and microbial consequences of MyD88 signaling. In wildtype and ft/ft mice, MyD88 was predominantly expressed in skin epithelia during homeostasis, whereas ft/ft^MyD88-/-^ mice developed spontaneous periocular skin inflammation. RNA-seq revealed upregulation of the IL-17 pathway in ft/ft^MyD88-/-^ inflamed skin. Flow cytometry identified Vγ4 ⁺ γδ T cells as the major source of IL-17A in ft/ft^MyD88-/-^ inflamed skin. We also discovered that ft/ft^MyD88-/-^ skin inflammation was associated with markedly downregulated lipid metabolism genes as well as sebaceous gland abnormalities histologically. Moreover, 16S rRNA gene sequencing demonstrated microbial dysbiosis in ft/ft ^MyD88-/-^ periocular skin, which drove skin inflammation as well as IL-17-producing γδ T cell infiltration. Our findings indicate a role for MyD88 in homeostatic control of sebaceous glands and suppression of dysbiosis-driven IL-17A-mediated inflammation in filaggrin-deficient skin. These insights advance our understanding of AD pathogenesis and may offer novel therapeutic strategies for patients with filaggrin mutations.

## Introduction

Atopic dermatitis (AD) is a chronic and relapsing skin disease that causes itchy, dry, and inflamed skin affecting up to 20% of children and 10% of adults and correlates to the increased risk of food allergy, asthma, and other immune-mediated inflammatory diseases [1]. AD is a heterogeneous disease that involves complex interactions between host immune dysfunction, skin microbiome alterations, and epidermal barrier disruption [2]. Epidermal barrier dysfunction is considered a predisposing factor in the development of AD, leading to transepidermal water loss, pH imbalance, and altered lipid composition [3,4], which makes AD skin more susceptible to penetration by allergens [5].

Filaggrin, encoded by the *FLG* gene, specifically binds to keratin intermediate filaments and is involved in epithelial differentiation [6]. Filaggrin loss-of-function mutations are associated with increased susceptibility for multiple skin and allergic disorders, including AD, ichthyosis vulgaris, asthma, food allergy, and rhinitis [7]. Notably, filaggrin loss-of-function mutations are the strongest genetic risk factor for AD, which results in increased epidermal barrier dysfunction [6]. In AD patients with filaggrin loss-of-function mutations, filaggrin expression is significantly reduced, highlighting the importance of this epidermal protein in barrier integrity and disease pathogenesis [8,9]. Furthermore, filaggrin loss-of-function mutations alter the skin microbiome [10–12] as well as IL-1 family cytokine expression in the epidermis [13,14], and promote an imbalance of effector rather than regulatory T cells during development [15], which contributes to the development of spontaneous and injury-induced skin inflammation [16,17]. However, the mechanisms that regulate skin homeostasis in filaggrin deficient skin are not entirely clear.

Myeloid differentiation factor 88 (MyD88) is an intracellular adaptor in the toll-like receptor (TLR) and interleukin-1 (IL-1) receptor family inflammatory signaling pathways [18]. In the skin, MyD88 promotes cutaneous host defense against *S. aureus* [19], contact hypersensitivity reactions [20], and inflammatory cytokine production [21]. Furthermore, MyD88 signaling is required for monocyte and macrophage activation and infiltration in imiquimod (IMQ)-induced psoriasis through IL-1β and IL-23 production [22]. In the context of filaggrin deficiency, the MyD88-associated cytokines, IL-1α and IL-1β, have been implicated in AD skin inflammation [13,16]. Conversely, MyD88 signaling also plays a crucial role in Treg immunoregulation by promoting microbial sensing as well as suppressing skin inflammation and food allergies [23–25].

Given the conflicting evidence, we set out to determine how MyD88 regulates skin homeostasis in mice with loss-of-function filaggrin mutations. In this study, we used filaggrin deficient (ft/ft) mice with and without MyD88 deficiency to explore the immunological and skin microbial consequences of MyD88 signaling in the context of filaggrin deficiency. Collectively, our findings have important implications for our understanding of the susceptibility of AD patients with filaggrin loss-of-function mutations as well as the development of new therapeutic strategies to restore skin homeostasis during skin inflammation.

## Materials and methods

### Mice

Sex- and age-matched 6-week-old mice were used for all experiments. ft/ft^MyD88-/-^ mice were generated as an in-house cross between a spontaneous loss-of-function point mutation in filaggrin (ft/ft) without the matted (ma) mutation and MyD88 deficient (MyD88^-/-^) mice on a BALB/c background, as previously described [16]. For reference, the ma mutation in the Tmem79/Matt gene is associated with spontaneous dermatitis in the original flaky tail mouse strain. All mouse strains were bred and maintained under the same specific pathogen-free conditions, with air-isolated cages at an American Association for the Accreditation of Laboratory Animal Care-accredited animal facility at Johns Hopkins University and handled according to procedures described in the Guide for the Care and Use of Laboratory Animals as well as Johns Hopkins University’s policies and procedures as set forth in the Johns Hopkins University Animal Care and Use Training Manual. For noninvasive skin imaging, mice were briefly anesthetized with 2–5% inhaled isoflurane. Euthanasia was performed by carbon dioxide inhalation, with death verified by lack of recovery after CO_2_ exposure followed by cervical dislocation. All animal experiments were approved by the Johns Hopkins University Animal Care and Use Committee (Approved protocol number: MO24M318).

### Inflammation area measurements

The periocular skin images were taken from 6-week-old mice with a digital camera. The skin inflammation area (cm^2^) was measured by analyzing digital photographs with ImageJ software (http://imagej.nih.gov/ij/), calibrated against a millimeter ruler as the reference scale.

### Histology, epidermal thickness, and sebaceous gland measurements

The periocular skin biopsy specimens were placed in 10% formalin and paraffin embedded. Skin cross-sections (4 μm) were prepared on charged glass slides and stained with hematoxylin and eosin (H&E) by the Johns Hopkins Reference Histology Laboratory according to clinical specimen guidelines. Skin sections were digitally scanned using a NanoZoomer XR (Hamamatsu, Japan) and were viewed and exported using NDP.view 2 software (Hamamatsu, Japan). At least 10 fields per mouse were measured for epidermal thickness measurements by using ImageJ software.

For sebaceous gland histology analysis, sebaceous gland area was manually annotated using the freehand region annotation tool. Each sebaceous gland lobe was considered as an individual unit, and the area of each sebaceous gland lobe was measured as the histologically-evident contiguous area of lipid-laden sebocytes. Sebaceous gland count was determined by counting the number of individual sebaceous gland lobule annotations, which was then normalized to the length of the epidermis in the respective skin biopsy sample, as measured using the freehand line annotation tool in the NDP.view2 software.

### Immunofluorescence microscopy

The periocular skin sections were performed on deparaffinized histologic sections following heat-mediated antigen-retrieval in TintoDeparaffinator citrate (Bio SB). Sections were washed with PBS-T (PBS + 0.5% Triton X100), blocked with PBS-T containing 10% goat serum (blocking buffer) for 1 hour at room temperature, then incubated overnight with the MyD88 primary antibody (1 µg/ml; MA5–35251; Invitrogen) in blocking buffer. The following day, stained slides were washed with PBS-T and incubated for 1 hour with goat anti-rabbit secondary antibody (2 µg/ml; A11034; Invitrogen). Mouse slides were mounted using ProLong™ Gold Antifade Mountant with DNA Stain DAPI (Invitrogen). Fluorescent images were taken at 400x magnification (Olympus VS200).

### Immunofluorescent quantification

MyD88 intensity was determined by first delineating the epidermal area then measuring the Raw Integrated Density (Epidermal area × Sum of all pixel values) using ImageJ software. Each data point represents at least 5 fluorescent images per mouse.

### RNA-seq analysis

The periocular skin biopsies of ft/ft and ft/ft^MyD88-/-^ mice were excised and snap-frozen in liquid nitrogen and stored in −80°C for RNA extraction. The protocols for RNA extraction, purification, library preparation, and cDNA sequencing are as previously described [26,27]. Briefly, total RNA was processed for high-throughput sequencing using the Illumina TruSeq mRNA Sample Prep v2 kit (catalog no. RS-122–2001 and RS-122–2002). mRNA was generated from approximately 0.1–3.0 μg of total RNA per sample by polyA purification, following converted to cDNA using random primers and reverse transcriptase. Adaptor barcodes were added, and cDNA libraries were purified and enriched by PCR. Samples were run on a 50 cycle single end Illumina HiSeq 2000. FastQC was used to assess quality control parameters before and after read filtering. After read filtering and quality checks, reads were aligned with the HISAT algorithm to *the Mus musculus* GCF_000001635.26_GRCm38.p6 reference genome from the NCBI database and with tophat2 to align to reference genes. Differentially expressed genes (DEG) were determined between ft/ft and ft/ft^MyD88-/-^ groups with HTSeq using default parameters [28]. The gene annotation and pathway analysis of significantly differentially expressed genes (Benjamin-Hochberg adjusted p-value < 0.05) were performed on DAVID Functional Annotation Tool (NIH) using gene sets from the Kyoto Encyclopedia of Genes and Genomes. Raw and processed RNA-seq data have been deposited at NCBI’s GEO database under accession number GSE301391 (https://www.ncbi.nlm.nih.gov/geo/query/acc.cgi?acc=GSE301391).

### RNA isolation and quantitative real-time PCR

The periocular skin biopsies were snap-frozen in liquid nitrogen and homogenized (Pro200 Series homogenizer; Pro Scientific) in TRIzol reagent (Thermo). RNA was extracted by using a Direct-zol RNA-Mini Prep kit (Zymo Research), followed by a High-Capacity RNA-to-cDNA Kit (Applied Biosystems) to synthesize cDNA according to the manufacturer’s protocol. Quantitative real-time PCR (qPCR) was performed with 100 ng of cDNA mixed with commercial TaqMan primers and TaqMan Gene Expression Master Mix (Applied Biosystems) using a CFX96 Real-Time PCR system (Bio-Rad). Commercially available primers and probes for mouse *Actb*, *Il17a*, *Il17f*, *Il6*, *Ifng*, *Il10*, *Il1b*, *Il23a*, *Il4*, *Tgfb1*, *Il12b*, *Il1a*, *Il13*, *Il22*, *Il5*, and *Tslp* were purchased from TaqMan (Thermo). Relative quantities of mRNA per sample were determined using the ΔΔC(T) formula.

### Flow cytometry

The periocular skin biopsies were excised, minced, and digested in RPMI containing 100 μg/mL DNase I (Millipore Sigma) and 1.67 Wunsch units/mL Liberase TL (Roche) for 1 hour at 37°C on a shaker at 70 rpm. Single-cell suspensions were acquired by filtering the digested samples through a 40-μm filter and grinding the remaining skin debris using a 3-ml syringe plunger. The cells were then washed once with RPMI and resuspended in PBS. Cells were then stained for 10 min at 4°C using antibodies resuspended in FACS buffer (PBS with 1% BSA and 5 mM EDTA) to detect the following cell surface markers: APC-vio770-anti-CD45 (REA737), PE-Vio770-anti-CD3 (REA641), VioBlue-anti-CD4 (REA604), PerCP-Vio700-anti-TCRγ/δ (REA633) (all from Miltenyi), and FITC-anti-TCR Vγ4 (UC3-10A6; BioLegend). Fc receptor binding was blocked using CD16/32 TruStain FcX (BioLegend). Intracellular staining was performed by incubating 1 × 10^6^ cells per well in RPMI supplemented with 10% FBS, 2 mmol/L glutamine, 100 U/mL penicillin, 100 mg/mL streptomycin, and cell stimulation cocktail (phorbol 12-myristate 1-acetate and ionomycin) plus protein transport inhibitors (brefeldin A and monensin; eBioscience) for 4 hours at 37°C. The cell viability was analyzed by staining with Viobility Fixable Dye (Miltenyi) for 15 min at room temperature, followed by staining intracellular mAbs using APC-anti-IL-17A (REA183), and APC-anti-IL-17F (REA666) (all from Miltenyi). Cell acquisition was performed on a MACSQuant Analyzer 16 flow cytometer (Miltenyi), and data were analyzed with MACSQuantify software (Miltenyi). Cell types were defined by flow cytometry according to the following gating strategies (S4 Fig): Total T cells were identified from the CD45 + CD3 + population from live cells, and CD4 + T cell and γδT cell populations were identified by their respective T-cell receptor CD4+ and γδT+ surface markers. Vγ4 + γδT + T cells were identified from the γδT+ cell population. Cytokine-producing cells were gated on their respective surface markers and either IL-17A or IL-17F.

### Topical antibiotic administration

The ft/ft^MyD88-/-^ mice were treated daily with topical Neosporin (Johnson & Johnson) or vehicle (white petroleum) for 7 days.

### Periocular skin swab samples collection and plating

Samples for microbiome sequencing were collected and processed similarly to those described previously [16]. Briefly, Catch-All Sample Collection Swabs (Epicentre) were saturated in sterile lysis buffer (20 mmol/L Tris [pH 8.0], 2 mmol/L EDTA, and 1.2% Triton X-100) before sample collection. The premoistened swabs were rubbed vigorously on the periocular skin approximately 30 times or, for negative controls, exposed to air without skin contact. Swabs were placed in tubes (Eppendorf Biopur) and stored at −80°C. Samples for culturing were collected by using a swab premoistened with PBS and rubbing the periocular skin using a sterile cotton swab and immediately plated onto 5% sheep’s blood agar (Hardy Diagnostics) and incubated for 24 hours at 37°C.

### DNA extraction and sequencing

DNA was extracted from swabs as previously described [16]. Briefly, swabs were incubated in Yeast Cell Lysis buffer (BioSearch Technologies) and ReadyLyse Lysozyme solution (Epicentre) at 37°C with shaking, followed by mechanical disruption using a TissueLyser (Qiagen). Samples were additionally incubated at 65°C for complete lysis, and cellular debris and proteins were removed using MPC Reagent (BioSearch Technologies). The remaining supernatants were processed using the PureLink Genomic DNA Kit (Invitrogen) and DNA product was eluted in DNA-Free PCR Water (MoBio). Control (air) swabs were identically extracted and sequenced with experimental samples, and no apparent contamination was detected. The bacterial 16S ribosomal RNA V1–V3 region was amplified by using primers 27F (5’-AGAGTTTGATCCTGGCTCAG) and 534R (5’-ATTACCGCGGCTGCTGG) with Illumina adapters (Illumina). PCR conditions were as follows: 2.5 μL of 10 × Buffer, 4 μL of dNAP mix, 0.25 μL of LA Taq Hot Start Polymerase (Takara Bio), 10 nmol of each primer, DNA-free PCR water, and 2.5 μL of DNA. Reactions were performed in duplicate for 30 cycles, combined, purified with Agencourt AmpureXP (Beckman), and quantified with the Quant-IT dsDNA Kit (Invitrogen). Equivalent amounts of amplicons were pooled together, purified with MinElute PCR Purification Kit (Qiagen), and sequenced on an Illumina MiSeq platform (Illumina) with 2 × 300-bp read length.

### Sequence analysis

Sequence reads were processed using DADA2 (v1.26.0) [29]. Forward and reverse 16S reads were filtered and trimmed according to the following parameters: truncLen = c(260,260), maxN = 0, maxEE = c(3,5), truncQ = 2. Taxonomic assignment was based on the Silva database (https://zenodo.org/record/4587955), with tryRC = TRUE parameter. In all other instances, default DADA2 parameters were followed (https://benjjneb.github.io/dada2/tutorial.html). A Phyloseq [30] object was built from the resulting ASVs and taxonomy. ASVs with fewer than 10 reads and samples with fewer than 10000 total reads were removed to minimize potential contamination. The top five most abundant phyla across ft/ft^MyD88-/-^ samples with verified collection dates and top abundant genera within each phylum were plotted. Beta diversity (Bray-Curtis dissimilarity) was calculated with vegan package. Principal coordinate analysis (PCoA) was conducted based on Bray-Curtis with vegan package. Data was visualized using the ggplot2 v3.5.1 (https://doi.org/10.32614/CRAN.package.vegan.; https://doi.org/10.1007/978-3-319-24277-4). The raw amplicon sequencing data are available in the NCBI BioProject database under project number PRJNA1276238. The codes utilized to generate Figs 5b, 5d and S1 is available on GitHub: https://github.com/skinmicrobiome/2025_Wu_MYD88.

### Statistical analysis

Data from single comparisons were analyzed by a two-tailed Student’s t test. Data from more than two comparisons were analyzed by a one-way ANOVA multiple comparisons test with Tukey correction. All statistical analyses were performed using Prism software version 10 (GraphPad). Data are presented as mean ± SEM, and values of P < 0.05 were considered statistically significant.

## Results

### MyD88 deficiency induces spontaneous skin inflammation in filaggrin-deficient mice

**Fig 1:**
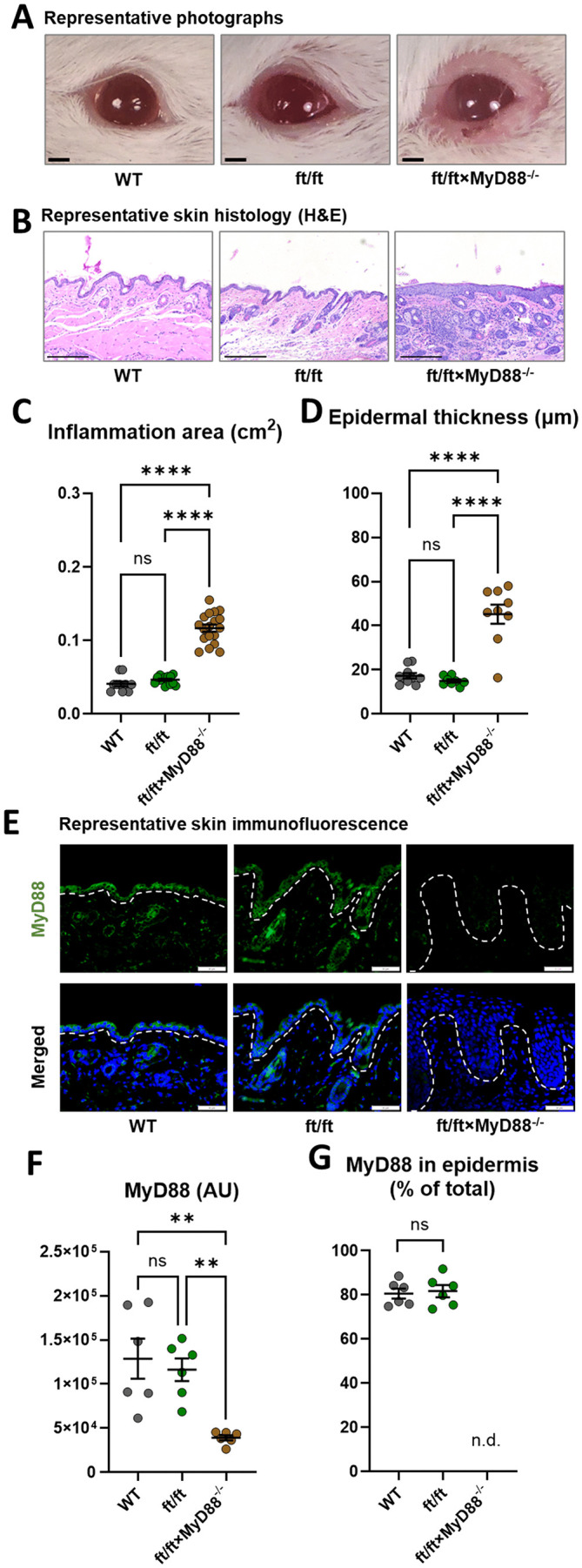
MyD88 restrains skin inflammation in filaggrin deficient mice. The periocular skin of WT, ft/ft, and ft/ft^MyD88-/-^ mice were examined. **(A)** Representative photographs of periocular skin. Scale bars, 1 mm. **(B)** Representative H&E periocular skin sections (scale bars, 200 µm). **(C)** Mean periocular skin inflammation area (cm^2^) ± SEM. **(D)** Mean epidermal thickness (µm) ± SEM. **(E)** Representative immunofluorescence of MyD88 (green) and DAPI (blue) on periocular skin. The dotted lines represent the epidermal-dermal junction. Scale bars, 50 µm. **(F)** Mean MyD88 intensity ± SEM. **(G)** Mean percentage of MyD88 intensity in epidermis ± SEM. n.d., not detected. Statistical significance was determined with one-way ANOVA. Data are combined from two independent experiments.

Filaggrin deficiency is the predominant genetic risk factor for AD [1]. We and others have shown that filaggrin-deficient skin exhibits increased IL-1α and IL-1β cytokines [13], which contribute to spontaneous and injury-induced skin inflammation [16,17], yet other studies have found that loss of MyD88 signaling promotes inflammatory responses at barrier tissues, including in filaggrin deficient skin [25]. However, the reasons for these conflicting reports on the role of MyD88 signaling in barrier homeostasis, especially in the context of filaggrin deficient skin, are not entirely known. Thus, we used mice with a spontaneous loss-of-function point mutation in filaggrin with the *ma* mutation removed (ft/ft mice) [16] and crossed them with MyD88 deficient mice on a BALB/c background (ft/ft^MyD88-/-^) [25]. As previously reported [25], ft/ft^MyD88-/-^ mice exhibited spontaneous periocular skin inflammation with complete penetrance by 6 weeks of age, whereas wildtype (WT) and ft/ft mice had no visible inflammation (Fig 1A and 1C). Similar to WT and ft/ft mice, prior studies have shown that MyD88-/- mice do not develop spontaneous skin lesions [25]. Histologically, ft/ft^MyD88-/-^ skin had increased epidermal thickening compared to WT and ft/ft mice (Fig 1B and 1D). Next, we determined which cells expressed MyD88 during skin homeostasis via immunohistochemistry. We found predominant expression in the epidermal cells in WT and ft/ft, but not ft/ft^MyD88-/-^ mice (Fig 1E). This was confirmed by quantification, which was similar between WT and ft/ft mice, but significantly higher compared to ft/ft^MyD88-/-^ mice (Fig 1F). Moreover, we discovered that the majority of the MyD88 expression (~80%) was localized to the epidermis in WT and ft/ft mice (Fig 1G). Collectively, these results suggest that MyD88 is predominantly expressed in the epidermis during skin homeostasis, which limited spontaneous inflammation in filaggrin-deficient mice.

### MyD88 deficiency triggers IL-17 production in filaggrin-deficient skin

**Fig 2:**
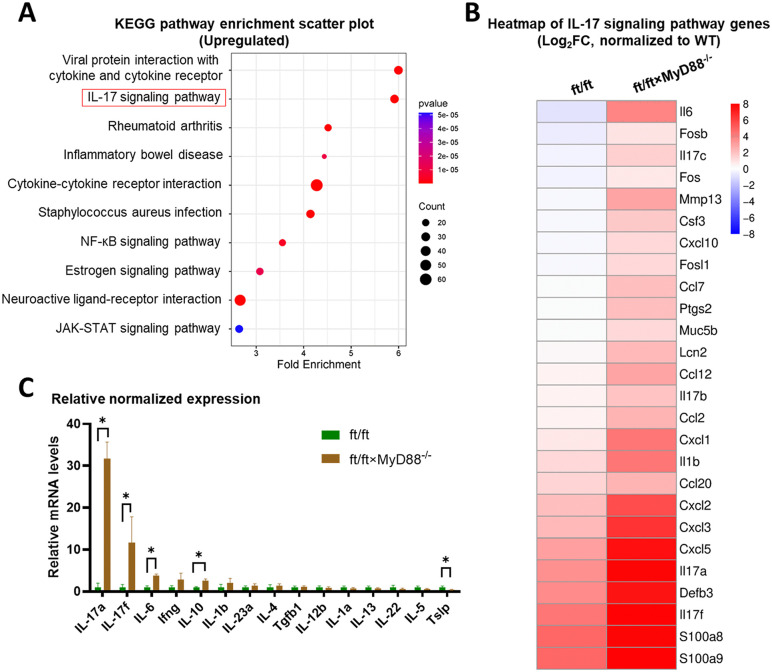
MyD88 restricted IL-17 signaling in filaggrin deficient skin. The periocular skin of ft/ft and ft/ft^MyD88-/-^ mice were evaluated by (A, B) RNAseq and (C) qPCR. **(A)** Upregulated KEGG pathway analysis from RNAseq of the ft/ft and ft/ft^MyD88-/-^ periocular skin. **(B)** Corresponding log2FC heatmap of the IL-17 signaling pathway in (A). Data are combined from 4 of WT, 4 of ft/ft, and 4 of ft/ft^MyD88-/-^ mice. **(C)** Relative normalized gene expression from qPCR. Data were normalized to *Actb*. Statistical significance was determined with two-tailed Student’s *t*-test.

To understand how MyD88 deficiency induced spontaneous inflammation in filaggrin deficient skin, we performed RNA-seq on the periocular skin of ft/ft and ft/ft^MyD88-/-^ mice. KEGG pathway analysis of up-regulated genes revealed that inflammatory pathways, including the IL-17 signaling, cytokine-cytokine receptor interaction, and NF-kappa B signaling pathways were increased in ft/ft^MyD88-/-^ mice as compared to ft/ft mice (Fig 2A). To determine the direction of the effect in relation to WT skin, we created a heat map of total genes and DEGs (S1 Fig) as well as a heat map of genes from the IL-17 signaling pathway identified by KEGG pathway analysis. Similar to prior reports [15], we observed significantly increased IL-17 pathway cytokines (e.g., *Il17a* and *Il17f*) in ft/ft as well as ft/ft^MyD88-/-^ mice compared to WT mice (Fig 2B). We confirmed the significantly induced expression of *Il17a*, *Il17f*, and *Il6* in ft/ft^MyD88-/-^ by qPCR (Fig 2C). The qPCR analysis also showed modest but significant differences in *Il10* and *Tslp*, but not other cytokines associated with Th17 skin inflammation (e.g., *Il1a*, *Il1b*, and *Il23a*) or Th2 inflammation (e.g., *Il4*, *Il5*, and *Il13*). Taken together, a robust IL-17 cytokine response was induced in the inflamed skin of ft/ft^MyD88-/-^ mice.

### MyD88 deficiency drives Vγ4+ γδT17 cell infiltration into filaggrin deficient skin

**Fig 3:**
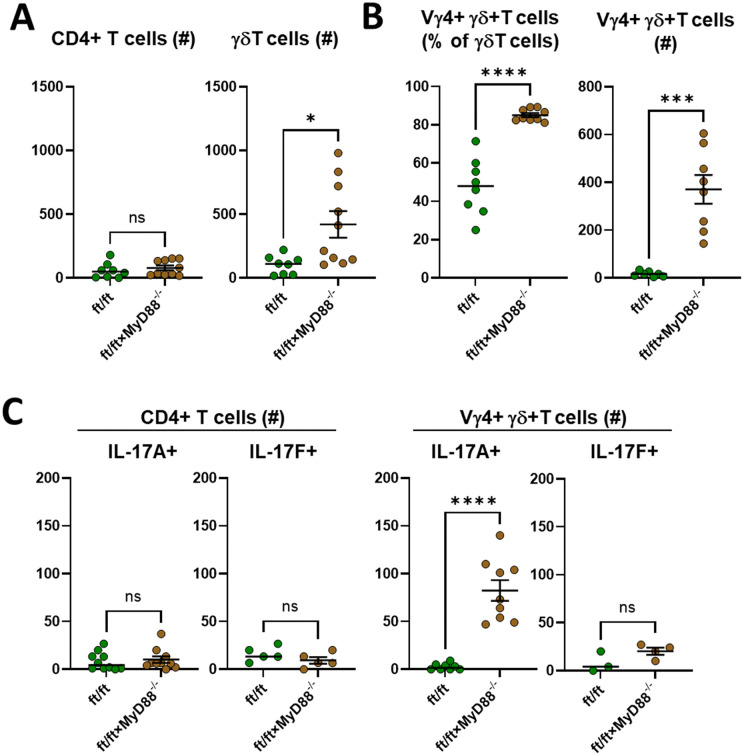
MyD88 limits Vγ4+ γδT17 cells in filaggrin deficient skin. The periocular skin from ft/ft and ft/ft^MyD88-/-^ mice were assessed by flow cytometry. **(A)** Mean number of CD4+ and γδT cells ± SEM. **(B)** Mean % of Vγ4+ γδ + T cells ± SEM from total γδT cells, and Mean number of Vγ4+ γδ+ T cells ± SEM. **(C)** Mean number of IL-17A- or IL-17F-producing CD4+ T cells and Vγ4+ γδ+ T cells ± SEM. **(A-C)** Statistical significance was determined with two-tailed Student’s *t*-test. Data are combined from or representative of two independent experiments.

We next set out to determine the cellular source of IL-17 in the inflamed skin of ft/ft^MyD88-/-^ mice. Since we and others have shown a predominant role of CD4 T cells and γδ T cells in IL-17 production during inflammation and infections [31–33], we examined these cell populations in the skin by flow cytometric analysis. We found a selective increase in γδ T cells, but not CD4+ T cells in ft/ft^MyD88-/-^ mice compared to ft/ft mice (Fig 3A). Next, we examined the dermal infiltrating Vγ4+ subset of γδT cells [33], which was markedly elevated in ft/ft^MyD88-/-^ mice compared to ft/ft mice (Fig 3B). Furthermore, Vγ4+ γδT cells, but not CD4+ T cells had a significant increase in IL-17A production compared to ft/ft mice (Fig 3C). However, neither CD4+ or Vγ4+ γδT cells showed an increase in IL-17F expression. Collectively, our results showed that MyD88 restricted IL-17A-producing Vγ4+ γδT cells (γδT17) in filaggrin-deficient skin.

### MyD88 regulates sebaceous gland homeostasis in filaggrin-deficient skin

**Fig 4:**
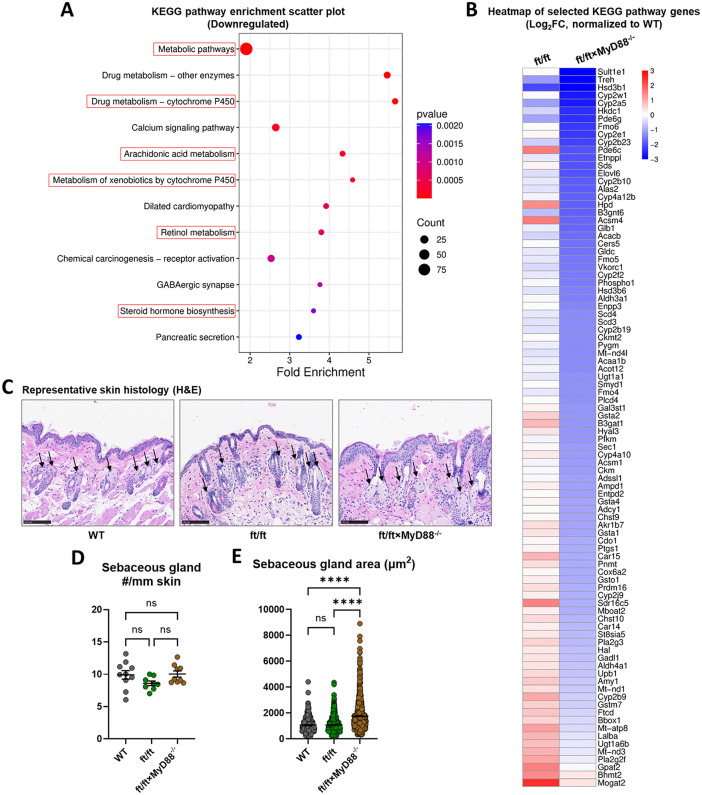
MyD88 controls sebaceous homeostasis. The periocular skin of ft/ft and ft/ft^MyD88-/-^ mice were evaluated by RNAseq and histology. **(A)** Downregulated KEGG pathway analysis from RNAseq of the ft/ft and ft/ft^MyD88-/-^ periocular skin. The pathways involved in the sebaceous gland homeostasis have been highlighted with red boxes. **(B)** Corresponding log_2_FC heatmap of the sebaceous gland-related pathway in (A). **(C)** Representative H&E periocular skin sections. Scale bars, 100 µm. Arrow indicates sebaceous gland. **(D)** Mean number of sebaceous gland ± SEM. **(E)** Mean area of sebaceous gland (µm^2^) ± SEM. Statistical significance was determined with one-way ANOVA. Data are combined from two independent experiments.

To uncover what pathways were dampened in ft/ft^MyD88-/-^ skin, we first performed KEGG pathway analysis on down-regulated genes identified in RNA-seq on the periocular skin of ft/ft and ft/ft^MyD88-/-^ mice. We found that pathways associated with sebaceous glands, including Metabolic pathways, cytochrome P450 metabolism [34], Arachidonic acid metabolism [35], and Steroid hormone biosynthesis pathways [36], were significantly suppressed in ft/ft^MyD88-/-^ mice compared to ft/ft mice (Fig 4A). This was especially apparent when the selected sebaceous gland associated pathway genes were normalized to WT mice (Fig 4B). To determine whether the transcriptional differences correlated with phenotypic differences, we performed histological analyses on WT, ft/ft, and ft/ft^MyD88-/-^ mice (Fig 4C). Although we observed no differences in the number of sebaceous glands in the skin between WT, ft/ft, and ft/ft^MyD88-/-^ mice (Fig 4D), we found that ft/ft^MyD88-/-^ mice had markedly larger sebaceous glands compared to WT and ft/ft mice (Fig 4E). These findings indicated that MyD88 promotes sebaceous gland homeostasis in filaggrin deficient skin.

### MyD88 restrains dysbiosis-mediated inflammation in filaggrin-deficient skin

**Fig 5:**
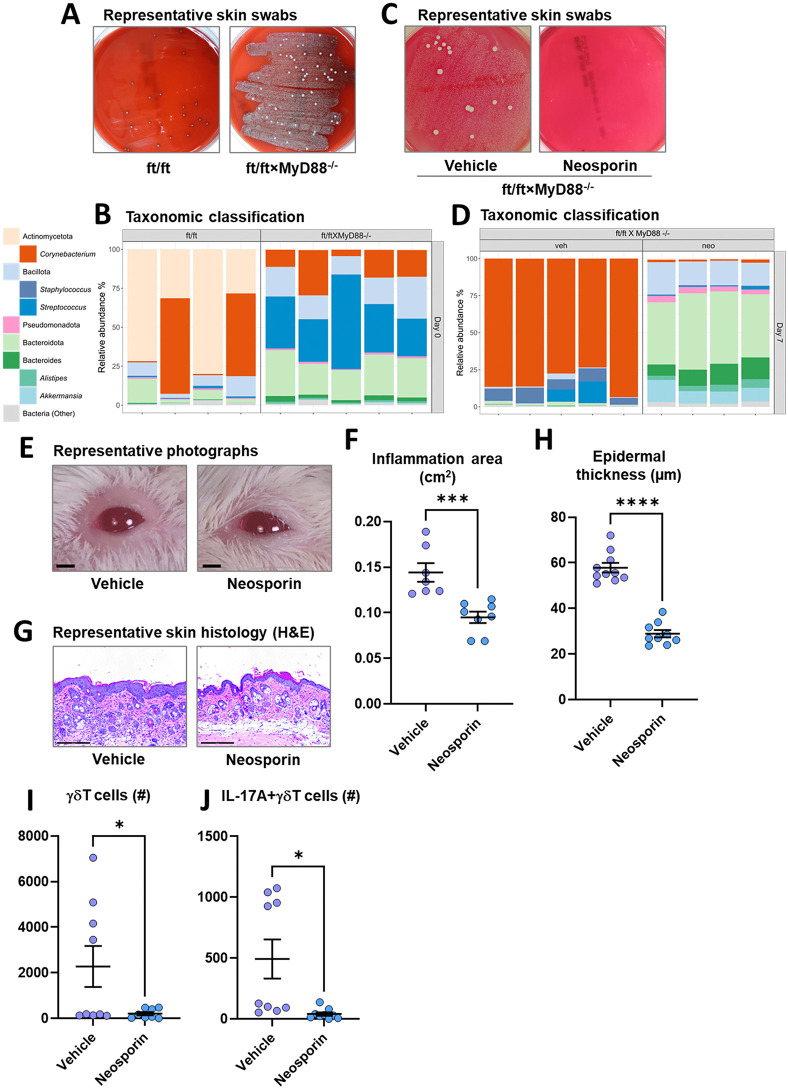
MyD88 restrains dysbiosis-mediated inflammation in filaggrin deficient skin. The periocular skin microbiome was examined in ft/ft and ft/ft^MyD88-/-^ mice. **(A)** Representative blood agar plates from ft/ft and ft/ft^MyD88-/-^ periocular skin swabs. **(B)** Taxonomic profiles of 16S sequencing from ft/ft and ft/ft^MyD88-/-^ periocular skin. **(C-K)** ft/ft^MyD88-/-^ periocular skin was treated daily for 7 days with vehicle or Neosporin. **(C)** Representative blood agar plates from skin swabs of vehicle or Neosporin treated ft/ft^MyD88-/-^ periocular skin. **(D)** Taxonomic profiles of 16S sequencing from vehicle or Neosporin treated ft/ft^MyD88-/-^ periocular skin. **(E)** Representative photographs of vehicle or Neosporin treated ft/ft^MyD88-/-^ periocular skin. Scale bars, 1 mm. **(F)** Mean periocular skin inflammation area (cm^2^) ± SEM. **(G)** Representative H&E periocular skin sections (scale bars, 200 µm). **(H)** Mean epidermal thickness (µm) ± SEM. **(I)** Mean number of γδT cells ± SEM. **(J)** Mean number of γδT17 cells ± SEM. Statistical significance was determined with two-tailed Student’s *t*-test. Data are combined from two independent experiments.

Prior reports have shown that sebaceous glands are a key component in regulating the skin microbiota [37]. Thus, we hypothesized that the abnormal sebaceous glands in ft/ft^MyD88-/-^ mice triggered skin dysbiosis and subsequent spontaneous skin inflammation. To this end, we swabbed the periocular skin of ft/ft and ft/ft^MyD88-/-^ mice and plated onto sheep’s blood agar. We found a significant outgrowth of bacteria from ft/ft^MyD88-/-^ mice but not ft/ft mice (Fig 5A), suggesting a shift in the microbiome on ft/ft^MyD88-/-^ skin. To address the differences in the microbiome, we performed 16S rDNA sequencing of periocular skin swabs from ft/ft^MyD88-/-^ and ft/ft mice (Fig 5B). We identified dramatic differences in the microbiota, including decreased relative abundances of *Actinomycetota* and increased *Bacillota* and *Bacteroidota* in ft/ft^MyD88-/-^ compared to ft/ft mice, with a notable expansion of *Streptococcus* on the ft/ft^MyD88-/-^ skin. We next treated ft/ft^MyD88-/-^ mice topically with Neosporin or vehicle (white petroleum) daily for 7 days to determine whether the microbiome had a role in spontaneous skin inflammation. To confirm that we sufficiently ablated the skin microbiome, we first swabbed the periocular skin and plated onto sheep’s blood agar. As expected, we observed no growth from swabs of Neosporin-treated mice whereas vehicle-treated mice had significant outgrowth on the plates (Fig 5C). Furthermore, 16S rDNA sequencing showed significant differences in bacterial community composition, with marked reductions in *Staphylococcus* and *Streptococcus* relative abundances in Neosporin-treated mice (Fig 5D). A Principal Coordinate Analysis (PCoA) showed separation between untreated (day 0) ft/ft and ft/ft^MyD88-/-^ mice as well as between Vaseline and Neosporin treated mice (S2 Fig), reinforcing our plate culture and 16S population observations. Importantly, Neosporin treatment significantly reduced skin inflammation grossly, as measured by reduced inflammation area (Fig 5E and 5F) as well as histologically, as measured by reduced epidermal thickness compared to vehicle controls (Fig 5G and 5H). We also discovered that reduced inflammation in Neosporin-treated ft/ft^MyD88-/-^ mice correlated with reduced total γδ T cells and γδT17 cells in the skin (Fig 5I and 5J). Moreover, γδ T cells and γδT17 cell populations significantly correlated with inflammation area, with a trend towards correlation with epidermal thickness (S3 Fig). Taken together, MyD88 restrained the development of dysbiosis-mediated inflammation in filaggrin deficient skin.

## Discussion

Filaggrin loss-of-function mutations are a predominant genetic risk factor for AD by causing epidermal barrier dysfunction that facilitates allergenic sensitization and pathogen colonization [12,38]. In this study, we revealed that MyD88 signaling contributed to maintaining skin homeostasis in filaggrin-deficient mice by regulating the microbiota. These findings provide important insights into the role of MyD88 in the development of skin inflammation in AD patients with filaggrin loss-of-function mutations and potentially other filaggrin-related inflammatory skin disorders and allergic diseases.

We uncovered that MyD88 is predominantly expressed in the epidermis during skin homeostasis. This may help explain why sebaceous glands, which are derived from epidermal progenitor cells [39], were phenotypically and transcriptionally dysregulated in ft/ft^MyD88-/-^ mice. Furthermore, the alteration in sebaceous gland functional pathways provide a potential explanation for the dysbiosis in ft/ft^MyD88-/-^ mice, since sebaceous glands are reported to regulate the skin microbiota [37]. Our RNA-seq data showed decreased expression of genes involved in sebocyte lipid metabolism and differentiation. We noticed downregulation of several key lipogenic machinery genes, including *Elovl6*, which is involved in fatty acid elongation and whose depletion has been associated with increased lipid droplet area [40]; *Acacb*, which is involved in fatty acid synthesis and terminal sebocyte differentiation, and whose reduction is associated with sebaceous gland hyperplasia [41]; and *Mogat2*, which is involved in triglyceride synthesis and whose inhibition has been reported to increase intracellular triglyceride levels and enlarge lipid droplet area [42]. Therefore, the enlarged sebaceous glands in ft/ft^MyD88-/-^ mice may reflect abnormal sebocyte maturation, in which sebocyte progenitors continue to expand, while terminal maturation and lipogenic machinery are impaired. This may explain the enlarged sebaceous glands in ft/ft^MyD88-/-^ mice since sebocytes may not properly mature or discharge sebum. This is further reinforced by recent findings that human atopic dermatitis patients, which exhibit significant dysbiosis [43], have alterations in sebum lipids compared to healthy controls [44,45]. However, whether these clinical observations involve MyD88 signaling pathways warrants further interrogation. We also noted that spontaneous inflammation in ft/ft^MyD88-/-^ mice was localized to the periocular region. This localization may be partly explained by the differences in the periocular microbiome from other skin sites [46] and the increased scratching behavior in ft/ft^MyD88-/-^ mice [25]. Thus, our findings indicate a role for MyD88 in maintaining skin homeostasis between sebaceous glands and the skin microbiome in the context of filaggrin deficiency.

We also discovered that the skin inflammation was associated with increased γδT17 cells, but not Th17 cells. Comparably, γδ T cells were the major source of IL-17 to drive skin inflammation in response to *S. aureus* epicutaneous exposure [31]. Since γδ T cell recognition of the microbiota triggers IL-17 production [47,48], the permissible barrier in filaggrin deficient skin may contribute to aberrant γδT17 cell activation and loss of skin homeostasis. Similar to previous studies in the skin [49], we identified the dermal Vγ4 subset of γδT17 cells to be involved in cutaneous inflammation. The robust γδT17 cell response in the absence of MyD88 signaling was unexpected, since IL-1R signaling is thought to be required for γδT17 cell development [50]. This may be due to compensatory microbial- or host-derived ATP release, which induces IL-17 production in T cells [51,52]. Furthermore, prior studies have found that MyD88 is crucial for Treg immunoregulatory functions at barrier sites [23,24], including in the context of filaggrin deficient skin [53]. Given the importance of Tregs in discrimination between commensals and pathogens during early-in-life cutaneous exposure [54], T cells in filaggrin deficient skin may have a different sensitivity towards MyD88 signals from the skin microbiota that results in an increased susceptibility to spontaneous skin inflammation. To this point, filaggrin deficient skin is associated with skewing from regulatory to effector commensal-specific CD4 + T cells [55], indicating an abnormal relationship with the skin microbiota during homeostasis. Similarly, *FLG* null‐mutations limit the expansion of circulating effector and memory Tregs in AD patients [56]. Understanding the interactions between the skin microbiota, γδT17 development, and Treg responses in the context of filaggrin deficiency will be the focus of future work.

We found that MyD88 regulated the skin microbiota profile on filaggrin deficient skin, which shifted from *Actinomycetota* to *Bacillota* in the absence of MyD88 signaling, especially *Streptococcus*. Likewise, a greater abundance of *Bacillota* is observed in pre-pubescent children [57], which may explain the higher prevalence of AD in young children. Notably, *Streptococcus pyogenes* is the second most common cause of skin and soft tissue infection as well as systemic infections in AD along with *S. aureus* [58,59] and correlates with increased disease severity [60,61]. Previous studies have shown that MyD88 signaling provides protection against *S. pyogenes* infection and tissue damage [62], which may further explain the skew towards *Streptococcus* colonization in the absence of MyD88. We also found that Neosporin treatment alleviated skin inflammation, which was associated with a decrease in *Streptococcus* and *Staphylococcus* as well as an increase in *Bacteroidota*, *Pseudomonadota*, and *Akkermansia* populations. The observed increase in *Pseudomonadota* after Neosporin treatment may reflect a return to skin homeostasis, since *Pseudomonadota* are abundant in healthy human skin [63,64]. Elucidating the role of *Streptococcus* in AD skin inflammation warrants further investigation. In contrast, Hoff et al. [25] reported that the microbiota were not required for the persistence of skin lesions in Flg^ft/ft^ mice. This discrepancy may be due to differences in animal housing facilities, which have been shown to influence the microbiome [65].

Our study had several limitations. First, we did not deplete γδ T cells or IL-17A using genetic mouse models or blocking antibodies to evaluate their role in the development of spontaneous skin inflammation. Future work will aim to better elucidate the role of γδ T cells and IL-17A in skin homeostasis in the context of filaggrin deficiency. Second, while we observed skin microbiome alterations associated with skin inflammation, we did not identify the specific pathogens involved in the development of spontaneous skin inflammation. Both *S. aureus* and *S. pyogenes* colonization are known to be major predisposing factors for increased infections in AD [58]. Therefore, we cannot conclude if specific bacterial pathogens contribute to spontaneous skin inflammation in this study, and further research will be required to address this question. Third, since we used a global MyD88 knockout mouse strain on a filaggrin deficient background, we did not determine the specific cell type responsible for the inflammation phenotype in ft/ft^MyD88-/-^ mice. Although we observed predominant MyD88 expression in the epidermis during skin homeostasis, MyD88 signaling in immune cells, including Tregs, may also contribute to limiting inflammation as previously described [25]. Another limitation of our study is that we did not include MyD88-/- mice on a filaggrin-sufficient background in all experimental comparisons. However, previous studies reported that MyD88-/- mice do not develop spontaneous skin lesions or increased Il17a expression in the absence of filaggrin deficiency [25]. Future studies including this control in parallel will further clarify how filaggrin deficiency modifies the function of MyD88 signaling in skin homeostasis. Finally, this study was conducted using age- and sex-matched female mice, limiting the generalizability of our findings with respect to sex-based differences.

In summary, our findings indicate that MyD88 regulates sebaceous glands and restricts dysbiosis-driven γδT17 dysregulation in filaggrin-deficient skin. These results have implications in the development of therapies that target the skin microbiota and T cell dysregulation in patients with filaggrin loss-of-function mutations and AD skin inflammation.

## Supporting information

S1 Fig Differential gene expression (DEG) of RNA-seq. (**A**) Global heatmap of the ft/ft and ft/ft^MyD88-/-^ periocular skin. Data was presented as Log2-transformed. (**B**) The number of DEGs.
(DOCX)

S2 Fig Bacterial community structure. Principal coordinate analysis (PCoA) of beta diversity (Bray-Curtis dissimilarity) among different groups. Each point represents a sample, colored by group (D0, ft/ft and ft/ft^MyD88-/-^; D7, ft/ft^MyD88-/-^ veh and ft/ft^MyD88-/-^ neo). Ellipses represent the 95% confidence interval around the centroid of each group.
(DOCX)

S3 Fig The inflammation area correlates with the number of γδT17 cells. The periocular skin of vehicle or Neosporin-treated ft/ft^MyD88-/-^ mice were examined. The linear regression between inflammation area and γδT cell number **(A)** or γδT17 cell number **(B)**. The linear regression between epidermal thickness and γδT cell number **(C)** or γδT17 cell number **(D)**. Data are combined from two independent experiments.
(DOCX)

S4 Fig Gating strategy for IL-17A + Vγ4 cells. Representative flow gating strategy for T cells.
(DOCX)

S1 File Graphical abstract. Loss of MyD88 in filaggrin-deficient skin results in sebaceous gland dysfunction and microbial dysbiosis. The imbalance in the skin microbiota promotes skin inflammation, characterized by the expansion of IL-17A-producing Vγ4⁺ γδ T cells. Topical antibiotic treatment restores microbial balance and reduces inflammation, highlighting a critical role for MyD88 in maintaining skin homeostasis in the context of filaggrin deficiency.
(TIF)
