# Supplementary material for: MyD88 restricts dysbiosis-mediated inflammation in filaggrin deficient skin
Source: PLoS One. 2026 Aug 14;21(8):e0353968. doi: 10.1371/journal.pone.0353968 (PMC13475889; doi:10.1371/journal.pone.0353968)
Supplement: Supplementary file 1 [file pone.0353968.s001.docx]

**S1 Fig. Differential gene expression (DEG) of RNA-seq.** (A) Global heatmap of the ft/ft and ft/ft^MyD88‑/-^ periocular skin. Data was presented as Log2-transformed. (B) The number of DEGs.
