# Supplementary material for: MyD88 restricts dysbiosis-mediated inflammation in filaggrin deficient skin
Source: PLoS One. 2026 Aug 14;21(8):e0353968. doi: 10.1371/journal.pone.0353968 (PMC13475889; doi:10.1371/journal.pone.0353968)
Supplement: Supplementary file 2 [file pone.0353968.s002.docx]

**S2 Fig. Bacterial community structure.** Principal coordinate analysis (PCoA) of beta diversity (Bray-Curtis dissimilarity) among different groups. Each point represents a sample, colored by group (D0, ft/ft and ft/ft^MyD88‑/-^; D7, ft/ft^MyD88‑/-^ veh and ft/ft^MyD88‑/-^ neo). Ellipses represent the 95% confidence interval around the centroid of each group.
