# Supplementary material for: MyD88 restricts dysbiosis-mediated inflammation in filaggrin deficient skin
Source: PLoS One. 2026 Aug 14;21(8):e0353968. doi: 10.1371/journal.pone.0353968 (PMC13475889; doi:10.1371/journal.pone.0353968)
Supplement: Supplementary file 3 [file pone.0353968.s003.docx]

**S3 Fig. The inflammation area correlates with the number of γδT17 cells**. The periocular skin of vehicle or Neosporin-treated ft/ft^MyD88‑/-^ mice were examined. The linear regression between inflammation area and γδT cell number **(A)** or γδT17 cell number **(B)**. The linear regression between epidermal thickness and γδT cell number **(C)** or γδT17 cell number **(D)**. Data are combined from two independent experiments.
