# Supplementary material for: MyD88 restricts dysbiosis-mediated inflammation in filaggrin deficient skin
Source: PLoS One. 2026 Aug 14;21(8):e0353968. doi: 10.1371/journal.pone.0353968 (PMC13475889; doi:10.1371/journal.pone.0353968)
Supplement: Supplementary file 4 [file pone.0353968.s004.docx]

**S4 Fig. Gating strategy for IL-17A+ Vg4 cells.** Representative flow gating strategy for T cells.
